# Long-term effects of intravitreal bevacizumab and aflibercept on intraocular pressure in wet age-related macular degeneration

**DOI:** 10.1186/s12886-021-02076-1

**Published:** 2021-08-28

**Authors:** Mikael Kähkönen, Raimo Tuuminen, Vesa Aaltonen

**Affiliations:** 1 Department of Ophthalmology, Turku University Hospital, PO Box 52, 20521 Turku, Finland; 2 Department of Ophthalmology, University of Turku, Turku, Finland; 3 Helsinki Retina Research Group, Faculty of Medicine, University of Helsinki, Helsinki, Finland; 4 Department of Ophthalmology, Helsinki University Hospital, Helsinki, Finland

**Keywords:** Wet age-related macular degeneration, Anti-vascular endothelial growth factor, Bevacizumab, Aflibercept, Intraocular pressure

## Abstract

**Background:**

To evaluate the incidence of sustained elevation of intraocular pressure (SE-IOP) associated with intravitreal injections of anti-vascular endothelial growth factors (anti-VEGF) bevacizumab and aflibercept in patients with wet age-related macular degeneration (wAMD).

**Methods:**

A retrospective cohort study consisting of 120 eyes from 120 patients with anti-VEGF treatment for wAMD. Three different anti-VEGF groups were considered: i) 71 cases receiving bevacizumab only, ii) 49 cases receiving bevacizumab before switch to aflibercept, iii) 49 cases after switch to aflibercept. 120 uninjected fellow eyes served as controls. SE-IOP was defined as an increase from baseline ≥5 mmHg on 2 consecutive follow-up visits. The incidence of SE-IOP was analysed using exact Poisson tests and survival analysis. The time course of IOP was evaluated with linear mixed effect modelling.

**Results:**

In total, 6 treated eyes (2.38% incidence per eye-year) and 9 fellow eyes (3.58% incidence per eye-year) developed SE-IOP, and survival analysis showed no statistically significant difference (*p* = 0.43). Furthermore, the incidence of SE-IOP did not differ between the three anti-VEGF groups. Comparing the injected eyes of patients under 70 years to those of patients over 70 years, there was a statistically significant difference in survival without SE-IOP (incidence of 16.7% vs 0.7%, respectively, *p* < 0.0001).

**Conclusion:**

Intravitreal anti-VEGF injections were not associated with sustained elevation of IOP. These results do not support the claim that repeated anti-VEGF injections could elevate IOP.

## Background

Age-related macular degeneration (AMD) remains the leading cause of irreversible blindness in people aged over 50 in western countries, the wet form (wAMD) being responsible for approximately 90% of blindness attributable to AMD [1]. The incidence of blindness from AMD has fallen significantly after the introduction of intravitreal injections of anti-vascular endothelial growth factors (anti-VEGF) for the treatment of wAMD [2, 3].

With the increasing number of patients receiving anti-VEGF therapy for extended periods [4], it is important to evaluate the long-term adverse effects of the available injections. Anti-VEGFs bevacizumab and aflibercept are both safe and effective in treating wAMD. Adverse ocular events are rare and include, for example, increased intraocular pressure (IOP), retinal detachment and endophthalmitis [5].

IOP spikes may occur after various intraocular procedures, and precautions are particularly important in patients with glaucoma [6–8]. The transient IOP elevation after anti-VEGF injections was first described by Hollands et al., and the phenomenon is well known today. In most people, IOP and mean ocular perfusion pressure recover in 30 min after the injection [9, 10].

Conversely, the long-term effects of anti-VEGF agents on IOP are debated. A report from the IRIS (Intelligent Research in Sight) Registry revealed a clinically and statistically significant sustained IOP rise overall and specifically in eyes receiving bevacizumab or ranibizumab, but not in eyes that received aflibercept [11]. The overall findings of the registry have been backed by multiple smaller studies [12–14].

In contrast, several reports have concluded that anti-VEGF therapies do not have a significant long-term effect on IOP [15–18]. An analysis of the VIEW (VEGF Trap-Eye: Investigation of Efficacy and Safety in Wet AMD) 1 and 2 studies showed a slight decrease in mean IOP in eyes receiving intravitreal aflibercept for 96 weeks while there was no such decrease in the ranibizumab group. The study also demonstrated a significant decrease in the incidence of elevated IOP when comparing aflibercept to ranibizumab [19]. In a recent meta-analysis of 46 studies investigating intravitreal bevacizumab, ranibizumab and aflibercept, IOP normalized 1 week after the injection and no significant change in IOP was found for longer time-intervals [20]. It remains inconclusive whether intravitreal anti-VEGF therapy leads to an elevated risk of sustained IOP rise.

The purpose of this real-world study was to evaluate the incidence of sustained elevation of IOP (SE-IOP) and the IOP kinetics in wAMD patients that were treated either with bevacizumab only or switched to aflibercept.

## Methods

### Study design and patients

This retrospective cohort study included 120 eyes from 120 patients with intravitreal anti-VEGF treatment for wAMD and 120 fellow eyes which were not treated with any intravitreal anti-VEGF agent (controls). Of the treated eyes, 71 received bevacizumab only (non-switched patients), while 49 received bevacizumab as first-line treatment and were later switched to aflibercept as per the treatment protocol (switched patients). Three study groups were formed: i) 71 patients receiving bevacizumab only, ii) 49 patients receiving bevacizumab before switch to aflibercept, iii) the same 49 patients receiving aflibercept after the switch.

The patients were treated at Turku University Hospital between June 2011 and July 2019, and they were identified through the corresponding diagnosis (International Classification of Diseases [ICD]: diagnosis H35.31 and procedure code CKD05). Patients were included in this study if they received bevacizumab only or bevacizumab and aflibercept consecutively in one eye for wAMD and they were anti-VEGF treatment-naive in both eyes. The study followed the guidelines of the Declaration of Helsinki.

Patient records and prescription databases were searched for possible confounding conditions (established glaucoma and ocular hypertension), medications (IOP-lowering drugs) and treatments (cataract surgery, glaucoma surgery, iridotomy and laser trabeculoplasty). In the study, the definition of glaucoma was based on ICD coding: eyes were treated as glaucomatous if they were associated with any sub-code under the main code H40, which includes established glaucoma as well as ocular hypertension.

### Exclusion criteria

Exclusion criteria were ongoing anti-VEGF treatment in the fellow eye, less than two follow-up visits in any study group, lack of meaningful IOP measurements in the fellow eye (e.g. ocular prosthesis, phthisis bulbi) and lack of a baseline IOP measurement in either eye. During the follow-up, if intravitreal anti-VEGF treatment was initiated in the fellow eye, or the patient dropped out of either the bevacizumab or aflibercept treatment protocol, data was included up to this event.

### Clinical evaluation

The patients were diagnosed with wAMD by typical findings in dilated fundoscopy and optical coherence tomography (OCT) (Heidelberg Spectralis, Heidelberg Engineering GmbH, Heidelberg, Germany): cystic macular edema (CME) and/or neuroepithelial detachment (NED) and/or pigment epithelial detachment (PED) and/or retinal hemorrhage. Fluorescein angiography was performed in cases when needed in differential diagnosis. Each visit included visual acuity testing and tonometry. Rebound tonometry (iCare® tonometer, Revenio Group, Vantaa, Finland) was the standard practice. Single measurements from alternate methods were excluded for the consistency of IOP data.

### Bevacizumab treatment protocol

Treatment of all patients started with three monthly injections of bevacizumab (induction phase), followed by a control visit 1 month after the last injection. If the patient had full response to bevacizumab (no CME, no NED, no new hemorrhage, no PED increase), the treatment was continued with three subsequent injections of bevacizumab with 2-month (8 weeks) intervals. The patients were controlled after the first 2-month interval with OCT, and later, 2 months after each series of three bevacizumab injections (visual acuity, tonometry, dilated fundoscopy and OCT). Any CME, NED, new hemorrhage and PED increase was considered activity. If activity was observed in any control, the injection interval was tapered between 4 and 8 weeks, depending on the observed activity and response. The treatment always continued for a minimum of 15 months, followed by a 12-month observation period.

### Aflibercept treatment protocol

If a sufficient response was not achieved with six monthly bevacizumab injections, the treatment was switched to aflibercept modified treat-and-extend regimen (TER) protocol. In the aflibercept modified TER protocol, the first injection interval was 4 weeks, and subsequent injection intervals were increased by 2 weeks when no activity was observed (no CME, no NED, no new hemorrhage, no PED increase). If any activity was observed, the injection interval was decreased by 1 week. If activity was observed repeatedly (2 times) with the same injection interval, the longest interval with which there was no activity, was kept for a period of two consecutive injection series and then extended again.

Next injection interval was decided based on the OCT findings during each injection visit. Follow-up visits were planned as follows: after three injections with 4–8-week intervals; after two injections with 9–12-week intervals; after one injection with 13–16-week interval.

### Definition of SE-IOP

SE-IOP was defined as an increase from baseline ≥5 mmHg on at least 2 consecutive follow-up visits. The criterion was chosen to take into account variation in baseline IOP and to exclude non-sustained elevation of IOP.

### Statistical analysis

Descriptive statistics for continuous variables are presented using mean and standard deviation (SD) when variable is normally distributed, and median with first and third quartiles (Q1, Q3) otherwise. For categorical variables, frequencies and percentages are used. Normality of variables was assessed with Shapiro-Wilk test. Baseline characteristics between the three study groups were tested for statistically significant differences using analysis of variance (ANOVA) for normally distributed and Kruskal-Wallis test for non-normally distributed continuous variables. Chi-square test was used for categorical variables. For some of the following analyses, the groups were combined to increase the statistical power.

To investigate the rate of SE-IOP, eye-year incidence rates with confidence intervals were calculated with exact Poisson tests. Only the time to first event was considered. Corresponding exact mid-p adjusted *p*-values are also presented. A generalised linear mixed effects model was created to assess the clinical predictors for SE-IOP. The interaction between each potential predictor and the treatment status of the eye (whether it was a treated or a fellow eye) was analysed first separately and then all statistically significant interactions were combined in a multivariate model. Additionally, the Kaplan-Meier method was used to estimate survival curves, and comparisons were made between the treated and fellow eyes, and between the treated eyes of two age groups (< 70 and ≥ 70 years) using log-rank test.

In addition to the incidence of SE-IOP, the time course of IOP was analysed. Time from the beginning of treatment was converted to a categorical variable. To compare the time course of IOP between the treated and fellow eyes and between the three study groups, linear mixed effect models were created. Least squares means were obtained from the models and plotted, and the interactions between time from baseline and predictor variables (treated versus fellow eyes, study groups versus each other) were analysed using ANOVA.

Two-sided tests were used in all statistical analyses and *p*-values < 0.05 were considered statistically significant. Statistical analysis was performed using r version 4.0.1 (R Foundation for Statistical Computing, Vienna, Austria).

## Results

### Patient characteristics

Patient characteristics are summarized in Table 1. Between the groups, switched patients on aflibercept had a significantly longer follow-up and a higher cumulative number of injections compared to the other two groups. Non-switched patients on bevacizumab had a longer treatment interval compared to the other two groups. There were no statistically significant differences in baseline or last IOP between treated and fellow eyes (Table 1).

**Table 1:** Patient characteristics

	Non-switched patients, *n* = 71	Switched patients, *n* = 49		
	Bevacizumab	Bevacizumab^a^	Aflibercept^a^	*p*-value
Sex (males), n (%)	29 (41)	15 (31)	15 (31)	0.39
Age at first injection (years), mean ± SD	78.3 ± 7.6	76.7 ± 8.0	78.0 ± 7.9	0.51
Glaucoma, n (%)				
Treated eye	3 (4)	5 (10)	5 (10)	0.36
Fellow eye	4 (6)	5 (10)	5 (10)	0.57
Pseudophakia, n (%)				
Treated eye	33 (46)	15 (31)	23 (47)	0.16
Fellow eye	31 (44)	17 (35)	21 (43)	0.58
Baseline IOP (mmHg), median (Q1; Q3)				
Treated eye	13 (12; 16)	13 (10; 15)	13 (11; 15)	0.85
Fellow eye	14 (11; 16)	12 (11; 16)	14 (10; 17)	0.82
Last IOP (mmHg), median (Q1; Q3)				
Treated eye	13 (11; 15)	13 (11; 15)	12 (10; 14)	0.069
Fellow eye	12 (10; 15)	14 (10; 17)	13 (10; 15)	0.26
Follow-up (days), mean ± SD	495.8 ± 245.2	494.5 ± 351.4	700.2 ± 331.0	< 0.001
No. of injections, median (Q1; Q3)	10 (9; 14.5)	9 (6; 15)	15 (12; 21)	< 0.001
Injection interval (days), median (Q1; Q3)	41.7 (34.2; 49.0)	36.5 (34.0; 40.3)	35.1 (30.4; 55.5)	0.008

^a^Same individuals

During the follow-up, glaucoma treatment was enhanced for one patient with bilateral selective laser trabeculoplasty and an additional IOP-lowering drug for the treated eye (Table 1, switched patients, bevacizumab). Another patient was treated for bilateral narrow-angle glaucoma with an IOP-lowering drug bilaterally, followed by a bilateral laser iridotomy (Table 1, switched patients, bevacizumab). This was the only patient diagnosed with glaucoma newly during the follow-up. None of the patients were subjected to glaucoma surgery. Patients without glaucoma did not use any IOP-lowering drugs or receive any other IOP-lowering treatments during the follow-up.

### Incidence of sustained elevation of intraocular pressure

Table 2 represents the eye-year incidence in each group and the difference between treated and fellow eyes. The incidence of SE-IOP was comparable between treated and fellow eyes among patients on bevacizumab only, and switched patients both during bevacizumab and during aflibercept administration (Table 2). Furthermore, using the treated eye of non-switched patients on bevacizumab as a reference, the treated eye of neither switched patients on bevacizumab (*p* = 1.00, Table 2) nor switched patients on aflibercept (*p* = 0.38, Table 2) showed a statistically significant difference in the eye-year incidence of SE-IOP.

**Table 2:** Incidence of SE-IOP

	Number of eyes	Eyes with SE-IOP	Eye-year incidence, % (95% CI)	*p*-value
Non-switched patients				
Bevacizumab				
Treated eye	71	3	3.17 (0.65; 9.26)	0.69
Fellow eye	71	2	2.11 (0.26; 7.63)	ref
Switched patients				
Bevacizumab				
Treated eye	49	2	3.09 (0.37; 11.18)	0.46
Fellow eye	49	4	6.17 (1.68; 15.79)	ref
Aflibercept				
Treated eye	49	1	1.07 (0.03; 5.98)	0.37
Fellow eye	49	3	3.28 (0.68; 9.57)	ref
All study groups				
Treated eye	169	6	2.38 (0.87; 5.17)	0.45
Fellow eye	169	9	3.58 (1.64; 6.8)	ref

**Fig. 1:**
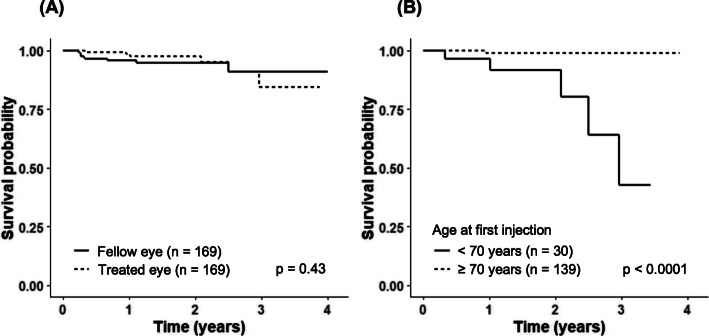
Kaplan-Meier survival curves for (**A**) SE-IOP in treated eyes versus fellow eyes and (**B**) in treated eyes of patients under 70 years versus those over 70 years. All study groups are pooled together. *P*-values were calculated with log-rank test

In total, 6 treated eyes (2.38% incidence per eye-year) and 9 fellow eyes (3.58%) developed SE-IOP, the difference being statistically non-significant (*p* = 0.45, Table 2). To illustrate the incidence of SE-IOP in treated and fellow eyes, Kaplan-Meier curves were drawn, showing no association between the development of SE-IOP and the treatment status of the eye (*p* = 0.43, Fig. 1A). Additionally, a generalized linear mixed effects model was created to assess the clinical predictors for SE-IOP during anti-VEGF therapy. All anti-VEGF groups were pooled together. The model revealed an inverse association between age and incidence of SE-IOP, younger age being a statistically significant risk factor (*p* = 0.018). In the univariate models, longer anti-VEGF treatment duration also appeared to be a risk factor (*p* = 0.036), but it was no longer statistically significant in the multivariate model (*p* = 0.140), whereas younger age remained a statistically significant risk factor (*p* = 0.016). A Kaplan-Meier curve is provided, showing this association (*p* < 0.0001, Fig. 1B). Sex, glaucoma, phakic status, baseline IOP, total number of injections and injection interval were not associated with SE-IOP during anti-VEGF therapy (data not shown).

### Time course of intraocular pressure

**Fig. 2:**
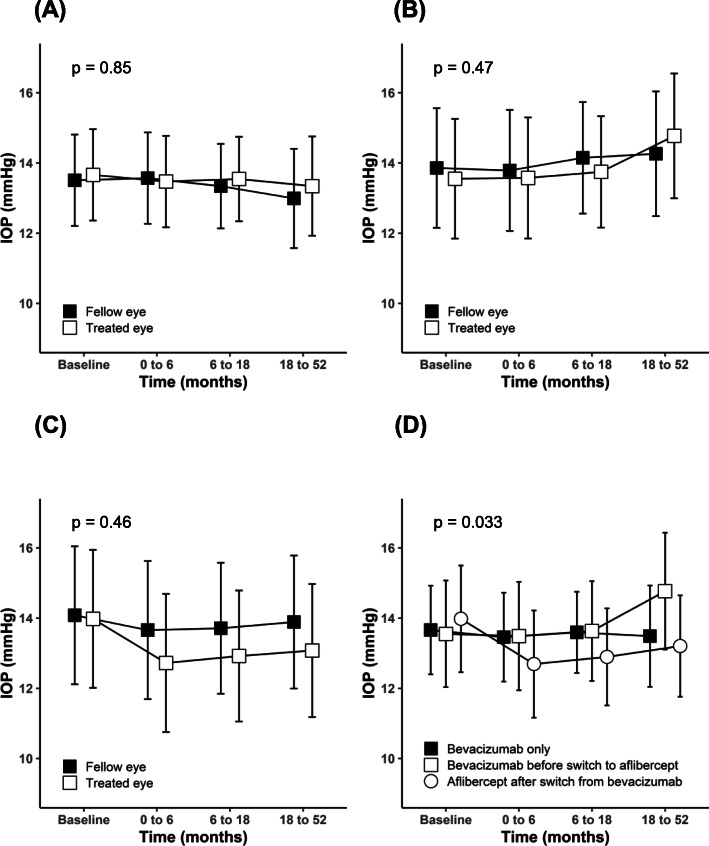
The time course of IOP (**A**) in treated versus fellow eyes during bevacizumab in non-switched patients, (**B**) bevacizumab in switched patients, (**C**) aflibercept in switched patients, and (**D**) in treated eyes only, comparing the three study groups. Plotted IOP data represent least squares means with 95% confidence intervals. Reported p-values describe the statistical significance of the interaction between time from baseline and treatment status (**A** - **C**), and time from baseline and study group (**D**)

Figure 2 demonstrates changes in IOP during follow-up. There were no differences in the time course of IOP between treated and fellow eyes in any group (Fig. 2A-C). Additionally, to illustrate the differences between the three groups, a line chart including only the anti-VEGF treated eyes from each group is provided. When comparing these treated eyes between the groups, a statistically significant difference was observed in the time course of IOP (*p* = 0.033, Fig. 2D). While IOP remained relatively constant in non-switched patients on bevacizumab, there was a slight increase in IOP in switched patients receiving bevacizumab and a slight decrease in switched patients on aflibercept. A comparison between switched patients on bevacizumab and switched patients on aflibercept showed statistical significance (*p* = 0.015, Fig. 2D).

## Discussion

The purpose of this study was to evaluate the incidence of SE-IOP and the time course of IOP in eyes receiving bevacizumab only or aflibercept after insufficient bevacizumab treatment response, and in their fellow eyes, in a real-life setting. A strict treatment protocol was followed, and only patients with unilateral wAMD were included. Pooling all study groups together, our results show a low eye-year incidence for SE-IOP, at 2.38% in treated eyes and 3.58% in fellow eyes. This incidence is in line with previous studies using similar criteria for SE-IOP [21].

Previous studies have had conflicting results on the long-term effects of anti-VEGFs on IOP. Reis et al. identified a statistically significant rise in the incidence of SE-IOP when comparing injected eyes to control eyes (7.47% versus 0.93%) [13]. Another controlled study by Wehrli et al. reported a 0.51% eye-year incidence in the treated eye and 1.00% in the fellow eye, a statistically non-significant difference [15]. Our results are in accordance with their conclusions that the difference in the incidence of SE-IOP between treated and fellow eyes is non-significant.

Reis et al. and Bilgic et al. showed that the incidence of SE-IOP is associated with older age and pre-existing glaucoma among other clinical predictors [12, 13]. Various potential predictors were analysed in our study, including glaucoma status, baseline IOP, sex and injection interval, but only younger age was determined to be a risk factor for SE-IOP associated with anti-VEGF therapy. This might partly be explained by a slight positive correlation between age and injection interval in our sample, younger people receiving more intensive therapy. However, injection interval itself was determined to not be associated with the incidence of SE-IOP. Younger patients also had a slightly higher baseline IOP, which itself showed a weak association with higher incidence of SE-IOP in the univariate model, while not quite being statistically significant. It should be noted that this effect might have been suppressed by some of the eyes with higher baseline IOP regressing towards the mean as the baseline only consisted of one measurement per patient.

Glaucoma treatment was changed bilaterally for two patients during the follow-up. Of these treatment modifications, one was symmetrical and one asymmetrical between the treated and fellow eyes. Therefore, the glaucoma treatment modifications are not likely to have any significant confounding effect. Furthermore, as the treatment for other glaucoma patients remained unchanged during the follow-up, and only patients with glaucoma or ocular hypertension received any IOP-lowering treatments, we conclude that IOP-lowering treatments are not significant confounders.

Our results are in line with previous reports in that bevacizumab seems to cause slightly higher IOP values than aflibercept [11, 18, 22]. The findings of Freund et al. demonstrating a slight decrease in IOP in eyes receiving aflibercept [19] are also supported by the present study. The reason for this favourable effect of aflibercept remains unknown. Unlike bevacizumab that only binds VEGF-A, aflibercept traps other VEGF family members too, including VEGF-B and placental growth factor (PlGF). Moreover, it has a markedly higher affinity for VEGF-A [23]. VEGF receptors expressed in the human trabecular meshwork tissue [24] could mediate unknown effects of VEGF-B and PlGF. It has also been hypothesized that silicone oil and protein particles found in anti-VEGF drugs might obstruct the outflow pathway [25], and this effect could in theory differ between the drugs. The researchers concluded that while bevacizumab and aflibercept are similar in average product quality, repackaged bevacizumab does show varying levels of contamination. Thirdly, repeated transient IOP spikes after injections might predispose the eye to SE-IOP via damaging the trabecular meshwork [26], although this cannot yet explain why aflibercept seems less harmful as no studies have demonstrated a statistically significant difference in the short-term course of IOP after an injection with bevacizumab versus aflibercept. More research comparing the pharmacodynamics of these two drugs are needed.

A notable limitation of this study was that the patients received aflibercept only after attempted therapy with bevacizumab. Because of this, it is possible that some of the effects seemingly caused by aflibercept resulted instead from the discontinuing of bevacizumab. However, the difference between the drugs remains. Additionally, the relatively small sample size may have decreased the ability to detect statistically significant effects some other researchers have demonstrated. The known systemic effects of intravitreal anti-VEGF injections [27] could also explain why no differences between the treated and fellow eyes were observed as the fellow eye might not be a true control.

## Conclusion

To summarize, our results show a low incidence of SE-IOP during anti-VEGF treatment, and no IOP-related effects attributable to the injections were seen. Younger patients might benefit from a closer follow-up of IOP. Some of these results conflict with past literature, and further controlled studies with larger sample sizes are still needed to investigate the possible long-term effects intravitreal anti-VEGF injections have on IOP.

## Data Availability

The datasets analysed during the current study are not publicly available in order to protect patient identity and confidentiality, but they are available from the corresponding author on reasonable request.

## Abbreviations

AMD: Age-related macular degeneration
Anti-VEGF: Anti-vascular endothelial growth factor
CME: Cystic macular edema
ICD: International Classification of Diseases
IOP: Intraocular pressure
NED: Neuroepithelial detachment
OCT: Optical coherence tomography
PlGF: Placental growth factor
PED: Pigment epithelial detachment
SE-IOP: Sustained elevation of intraocular pressure
TER: Treat-and-extend regimen
wAMD: Wet age-related macular degeneration
